# Study on the Ingredient Proportions and After-Treatment of Laser Sintering Walnut Shell Composites

**DOI:** 10.3390/ma10121381

**Published:** 2017-12-02

**Authors:** Yueqiang Yu, Yanling Guo, Ting Jiang, Jian Li, Kaiyi Jiang, Hui Zhang

**Affiliations:** 1 College of Mechanical and Electrical Engineering, Northeast Forestry University, Harbin 150040, China; yuyaoqiang.1228@163.com (Y.Y.); jiangting1112@163.com (T.J.); lijian499@163.com (J.L.); zh1226419340@163.com (H.Z.); 2 Research and Development Center of 3D Printing Material and Technology, Northeast Forestry University, Harbin 150040, China; 3 College of Engineering and Technology, Northeast Forestry University, Harbin 150040, China; jevons007@163.com

**Keywords:** waste, walnut shell, 3D printing, selective laser sintering, after-treatment

## Abstract

To alleviate resource shortage, reduce the cost of materials consumption and the pollution of agricultural and forestry waste, walnut shell composites (WSPC) consisting of walnut shell as additive and copolyester hot melt adhesive (Co-PES) as binder was developed as the feedstock of selective laser sintering (SLS). WSPC parts with different ingredient proportions were fabricated by SLS and processed through after-treatment technology. The density, mechanical properties and surface quality of WSPC parts before and after post processing were analyzed via formula method, mechanical test and scanning electron microscopy (SEM), respectively. Results show that, when the volume fraction of the walnut shell powder in the WSPC reaches the maximum (40%), sintered WSPC parts have the smallest warping deformation and the highest dimension precision, although the surface quality, density, and mechanical properties are low. However, performing permeating resin as the after-treatment technology could considerably increase the tensile, bending and impact strength by 496%, 464%, and 516%, respectively.

## 1. Introduction

Additive manufacturing (AM), commonly known as 3D Printing, is a technology combined with computer aided design, materials processing and molding. Based on digital model file, software and control system, three-dimensional objects are fabricated by successive layers made by laser sintering, fused deposition modeling [1,2], stereolithography [3], three-dimensional printing [4] and hot-pressing [5]. Selective laser sintering (SLS) is one of 3D printing technologies, which was put forward by C. R. Deckard [6] in his doctoral thesis at the University of Texas at Austin in 1988. Compared with other 3D printing technologies, SLS has some advantages such as no support is needed during manufacturing, materials can be reused, and parts have high precision [7]. Thus, SLS has been widely applied in industries such as automobile making, medical treatment, casting, aerospace and construction [8,9].

It is the feedstock of SLS that not only affect the development of SLS but also play a decisive role in the mechanical strength and surface quality of sintered parts. At present, feedstock for SLS are mainly focused on metal, ceramic, polymer and their corresponding composites [10,11,12,13,14,15,16,17,18,19]. However, because of rare species, lack structural diversity and high price, these materials do not meet the market demand and limit the development of SLS. Therefore, it is urgent to develop a sustainable, low-cost and environmentally friendly material for SLS. Professor Guo was the first devoted to developing natural, low-cost, recycled biomass material as the feedstock for SLS. Initial studies were mainly focused on single sintering experiment, forming process, and the development of composites such as aspen wood, rice husk and birch wood [20,21,22]. With more in-depth study on pine wood, bamboo and walnut shell, sintered parts of high dimensional precision, good surface quality and strength properties were obtained [23,24,25]. Different biomass powder particles have different geometry, distribution, physical and chemical properties, which have direct effect on the formability of sintering the composite. Compared to wood powder, bamboo powder and rice husk powder, the walnut shell powder has unique advantages such as wide source, easy to crush and good absorbability. In addition, approximately spherical walnut shell particles are helpful for powder spreading and dispersing, which are the basis of producing high-precision parts. Porous walnut shell particles have a good permeability, which is helpful to improve the mechanical strength of sintered parts.

Therefore, waste walnut shell was chosen as raw material, added in copolyester hot melt adhesive (Co-PES) matrix. Walnut shell composites (WSPC) powder of different content proportions was studied systematically. In addition, laser sintering mechanism and after-treatment technology were studied in-depth to assess the density, molding precision, mechanical property and surface quality of the WSPC parts and after-treatment parts. This lays a good basis on the molding precision, mechanical property and surface quality of the WSPC parts.

## 2. Materials and Methods

### 2.1. Experimental Materials

Walnut shell powder (approximately spherical particles, particle diameter range of 58–96 µm, and apparent density of 0.48 g/cm^3^) was obtained from food enterprise. Co-PES powder (particle diameter range of 0–58 µm, apparent density of 0.7 g/cm^3^, melted index of 30 g/10 min@160 °C and viscosity of 350 Pa·s@160 °C) was copolyester hot melt adhesive powder provided by Shanghai Tiannian Material Technology Ltd. (Shanghai, China). Industrial wax (block, melting point of 58.58 °C, oil addition of 0.32%, needle penetration of 16 mm, No. 30 color, No. 3 light stability, kinematic viscosity of 3.786 mm^2^/s, No. 1 odor) was a mixture of solid high paraffin provided by Jiangyin Chuanglin Chemical Ltd. (Guangzhou, China). Stearic acid (particle, melting point of 69.6 °C, boiling point of 232 °C, density of 0.94 g/cm^3^, decomposition temperature of 360 °C, flashing point of 220.6 °C) was a solid stearic acid provided by Hebei Delun Chemical Technology Ltd. (Shijiazhuang, China). Epoxy resin E-44 (transparent liquid, epoxy equivalent weight of 70–210 g/eq, softening point of 28–40 °C) was an epoxy resin adhesive provided by Nantong Xingchen synthetic material Ltd. (Nantong, China). Low molecular 651 polyamide resin (red brown viscous liquid, amine value of 400 ± 20 mgKOH/g, viscosity of 200–3000 cP at 40 °C), a good epoxy resin curing agent and toughening agent, was produced by Beijing Xiangshan joint additives factory (Beijing, China). Industrial alcohol (transparent liquid, alcohol content of greater than 99.9%, density of 0.791 g/cm^3^) was provided by Harbin Datong Chemical Ltd. (Harbin, China).

### 2.2. WSPC Powder Preparation

**Figure 1:**
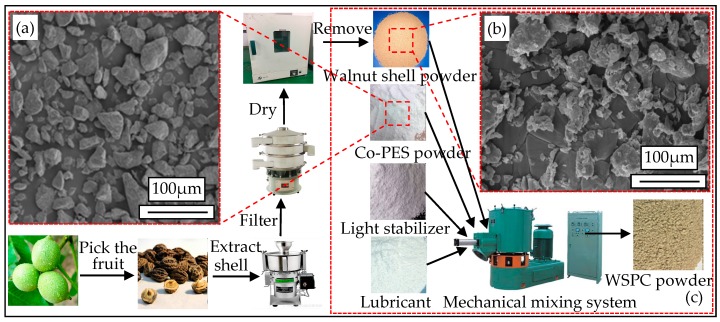
Walnut shell composites (WSPC) power preparation process: (**a**) copolyester hot melt adhesive (Co-PES) powder particles morphology; (**b**) walnut shell powder particles morphology; and (**c**) WSPC powder mechanical mixing process.

WSPC powder mainly consisted of walnut shell powder, Co-PES powder and micro-additive. The walnut shell waste was processed by crushing, rotating, polishing, steaming, washing and filtering, then, yellow-brown superficial porous particles were obtained. The microscopic morphology of the particles was shown in Figure 1a. In Figure 1b, Co-PES powder was white copolyester hot melt adhesive powder. The micro-additive mainly consisted of a small amount of light stabilizers and lubricants, which improved the mixing efficiency and laser sintering performance.

Before the preparation of WSPC powder, walnut shell powder was dehydrated for 3.5 h in an incubator of Beijing Longyuan Technology Ltd. (Beijing, China) at a temperature of 105 °C. During dehydration, the walnut shell powder was weighed at 1 h intervals until the mass kept constant. Then, the dried walnut shell powder was mixed with Co-PES according to specific formulas (shown in Table 1) using an SHR50A high-speed mixer from Zhangjiagang Hongji Machinery Ltd. (Dongying, China). To obtain preferable particle dispersion, a micro-additive was added during the mixing process. The powder mixture was mixed for 15 min below 30 °C at low-speed and then 5 min at high-speed. The mixed powder was taken out from the high-speed mixer and cooled naturally to obtain the WSPC powder for SLS. The preparation process is shown in Figure 1c. 

### 2.3. SLS Experiments and Post Processing

**Figure 2:**
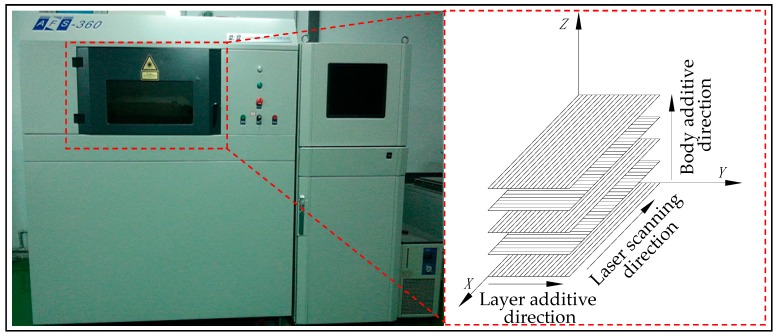
The equipment and building method.

SLS experiments: The sintering process of the WSPC parts with different ingredient proportions were conducted on an AFS-360 rapid prototyping equipment produced by Beijing Longyuan Technology Ltd. (Beijing, China). The equipment and building method are shown in Figure 2. The main process parameters are as follows: laser power of 14 W, scanning speed of 2000 mm/s, layer thickness of 0.1 mm, scan spacing of 0.2 mm, preheating temperature of 82 °C, and processing temperature of 75 °C.

**Figure 3:**
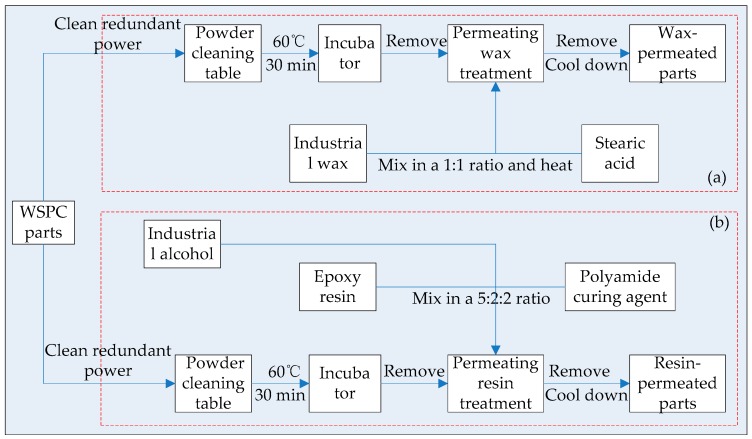
The post processing flow chart of WSPC parts: (**a**) permeating wax technology; and (**b**) permeating resin technology.

Post processing: The WSPC parts were removed from the AFS-360 rapid prototyping machine, and redundant unsintered powder was cleaned up thoroughly on a powder-cleaning table. Then, they were placed in an incubator of Beijing Longyuan Technology Ltd. at a temperature of 60 °C for 30 min. The post processing flow chart of WSPC parts is shown in Figure 3.

Permeating wax technology is shown in Figure 3a. The stearic acid was mixed with industrial wax in 1:1 ratio and heated to 70 °C. When the temperature kept constant, the WSPC parts were removed from the incubator and then immersed slowly in the melted mixed liquor using a hook, with an immersion time of about 5–10 s, and then slowly removed and placed on a filter paper which was used to absorb the redundant liquor on the surface of the parts. They were left to cool down, then the wax-permeated parts were obtained.

Permeating resin technology is shown in Figure 3b. The epoxy resin, polyamide curing agent and industrial alcohol were mixed in a ratio of 5:2:2, as resin mixed solution. The WSPC parts were removed from the incubator and then the surface was smeared with the resin mixed solution using a brush. When the permeation stopped, the redundant solution was cleared using a filter. After cool down, the resin-permeated parts were obtained.

### 2.4. Characterization

DSC: The WSPC powder with different ingredient proportions was analyzed using an American Pyris Diamond differential scanning calorimeter (PerkinElmer, Waltham, MA, USA). Testing parameters are as follows: the mass of every WSPC sample was 5 mg; heating rate was 10 °C/min; the range of testing temperature was 40–200 °C. DSC curves of the WSPC powder with different ingredient proportions were obtained.

SEM: As specimens were non-conductive, they were sputtered with gold first. The walnut shell powder, Co-PES powder, WSPC parts with different ingredient proportions, and cross sections of after-treatment bending parts were scanned using a FEI Quanta200 SEM produced by the Dutch company (Amsterdam, Netherlands). The powder and cross sections of specimens were observed. The SEM diagram of morphology of powder particles and the inner microstructures of the before and after post processing WSPC parts with different ingredient proportions were obtained.

Density: The mass and size of the WSPC parts and after-treatment parts were measured via an electronic balance and vernier caliper. Herein, the density ρ is calculated via Equation (1).

(1) $$\rho =\frac{W}{l\cdot b\cdot h}$$

where *W* is the mass of parts (g); *l* is the length of parts (mm); *b* is the width of parts (mm); and *h* is the thickness of parts (mm).

Mechanical test: Mechanical performances of the WSPC parts and after-treatment parts with different ingredient proportions were tested using a CMT5504 tensile testing machine of TMS System Ltd. and a TCJ-4 impact testing machine of Jilin Province Taihe Ltd. (Changchun, China). The testing standards are as follows: Tensile strength is determined in terms of the ISO527-2 Standard. Crosshead speed is 5 mm/min, and the gauge length is 50 mm. Three-point bending strength is determined in terms of the ISO178 Standard. Crosshead speed is 5 mm/min, and span length is 64 mm. U-notched impact strength is determined in terms of the ISO179-2 Standard. Pendulum impact power is 4 J, and span length is 60 mm. 

## 3. Results and Discussion

### 3.1. Thermal Analysis

Before the SLS of the WSPC powder with different ingredient proportions, the sintering window were determined via differential scanning calorimeter (DSC) to prevent them from warping deformation and hardening and improve the sintering performance of parts and the utilization ratio of powder. Therefore, the determination of the sintering window for amorphous polymers without melting point was great important.

**Figure 4:**
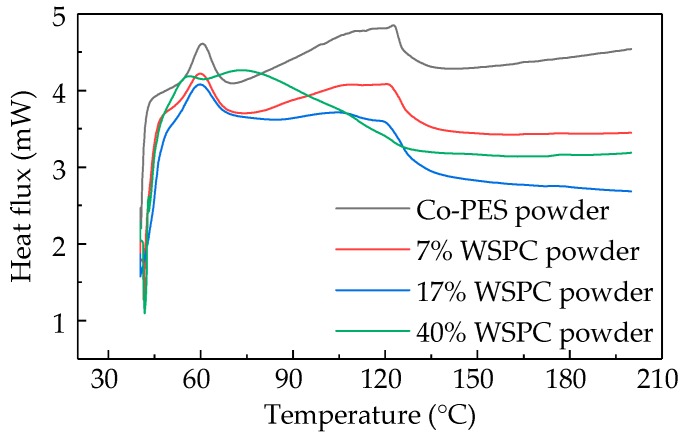
Differential scanning calorimeter (DSC) curves of the WSPC powder with different ingredient proportions.

DSC curves of the WSPC powder with different ingredient proportions are shown in Figure 4, which shows that the glass transition temperatures of Co-PES powder (0% walnut shell powder), 7% walnut shell powder, 17% walnut shell powder and 40% walnut shell powder were 57.48, 58.15, 59.72 and 61.05 °C, respectively. To determine the sintering window, the laser sintering experiment is needed. From the experiments, it can be observed that the WSPC powder with different ingredient proportions was completely hardening at 92, 93 , 95 and 103 °C, respectively. Thus, corresponding sintering window were (57.48, 92 °C), (58.15, 93 °C), (59.72, 95 °C) and (61.05, 103 °C), respectively. There was a heat accumulation phenomenon in the powder bed during sintering; thus, the preheating temperature and processing temperature of the WSPC powder bed with different ingredient proportions were 82 and 75 °C, respectively.

### 3.2. Laser Sintering Experiments

**Figure 5:**
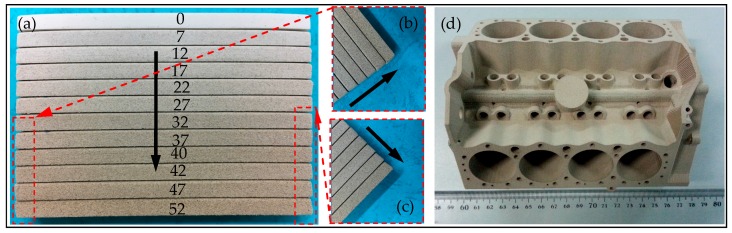
WSPC parts: (**a**) bending parts with different ingredient proportions; (**b**) left defect; (**c**) right defect; and (**d**) demonstration part.

In the process of SLS, the precision of sintering parts is a significant evaluation index of sintering material and sintering process. The WSPC parts with different ingredient proportions are shown in Figure 5. Figure 5a shows that the warping deformation of the WSPC parts is the largest when the volume fraction of the walnut shell powder is 0%. With an increase in walnut shell powder content, the warping deformation of the WSPC parts decreases. However, when the volume fraction of the walnut shell powder is 42%, the shape dimension deviation of the WSPC parts appears and then increases with an increase in walnut shell powder content, shown in Figure 5b,c. The temperature has a greater effect on Co-PES powder. Higher laser sintering temperature makes inner forming temperature of rapid prototyping machine decline quickly, which causes the warping deformation of the Co-PES parts. With an increase in walnut shell powder content, Co-PES powder decreases and the temperature has less effect on WSPC powder, thus the warping deformation of the WSPC parts decreases. However, when the volume fraction of the walnut shell powder reaches 42%, the friction increases among walnut shell powder particles, Co-PES powder and spreading roller during spreading powder, thus the shape dimension deviation of the WSPC parts appears and increases with an increase in walnut shell powder content. Therefore, the walnut shell powder content need not be too much. When the dimension of sintered parts is within a suitable range, the volume fraction of the walnut shell powder in the WSPC powder reaches 40%. V8 engine cylinder demonstration part is shown in Figure 5d. It shows that the light brown WSPC part has clear outline, good surface quality, and high shape precision, but poor mechanical strength for direct use in automobile industry. Thus, it is often used as demonstration part of new product development. The parts after post processing could have an enhanced mechanical strength and be used as wood pattern and investment casting.

### 3.3. Density of Parts

**Figure 6:**
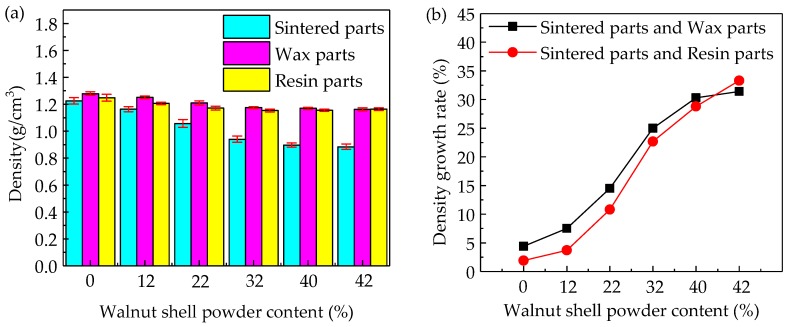
The density changes and growth rate curves of WSPC parts and after-treatment parts with different ingredient proportions: (**a**) density bar chart; and (**b**) density growth rate curves.

In the process of SLS, the density of sintering parts is one of criteria evaluating the performance. The density changes and growth rate curves of WSPC parts with different ingredient proportions are shown in Figure 6. Figure 6a shows that, with an increase in walnut shell powder content, the density of the WSPC parts and keeps constant finally. The mainly reason is that, with an increase in walnut shell powder content, Co-PES powder content decreases and it can not wrap and bonds the walnut shell powder, which results in the inner holes of WSPC parts. The holes become bigger and more with an increase of the walnut shell powder, thus the density decrease continuously. The density of after-treatment parts also decreases then tend to constant. However, compared with the WSPC parts, the density of after-treatment parts increases and the growth rate is quickly, shown in Figure 6b. The mainly reason is that, with a decrease in walnut shell powder content, Co-PES powder content increases and it can wrap and bond the walnut shell powder adequately, which results in the fewer inner holes and higher density of WSPC parts. After the parts are processed via permeating wax and permeating resin, it is hard for the wax mixed liquid and resin mixed liquid to permeate to the interior of the parts, thus the influence is less. However, with an increase in walnut shell powder content, Co-PES powder content decreases, resulting in bigger and more inner holes of WSPC parts. After the parts are processed via permeating wax and permeating resin, the wax mixed liquid and resin mixed liquid fill and solidify the holes of parts, which makes the density of the parts higher. However, when the walnut shell power content reaches a specific value, the inner permeating liquid wax and resin of WSPC parts is saturated, thus the density of the parts keeps constant. Therefore, the density of wax-permeated parts and resin-permeated parts decrease continuously and tend to constant finally. The density growth rate *η* was calculated via Equation (2).

(2) $$\eta =\frac{\rho_{1}-\rho_{0}}{\rho_{0}}\times 100\%$$

where *η* is density growth rate (%); $\rho_{0}$ is density of WSPC parts (g/cm^3^); and $\rho_{1}$ is density of after-treatment parts (g/cm^3^).

### 3.4. Microstructure

**Figure 7:**
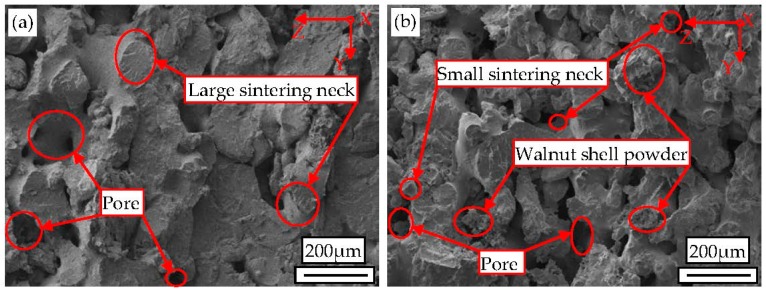

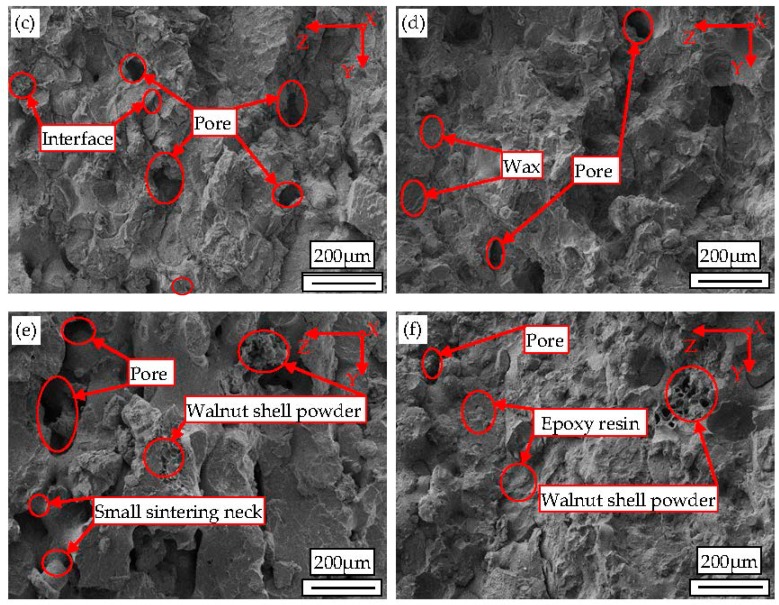
The scanning electron microscopy (SEM) diagrams of cross-sections of WSPC parts and after-treatment parts with different ingredient proportions: (**a**) WSPC parts (22% walnut shell powder); (**b**) WSPC parts (40% walnut shell powder); (**c**) WSPC wax-permeated parts (22% walnut shell powder); (**d**) WSPC wax-permeated parts (40% walnut shell powder); (**e**) WSPC resin-permeated parts (22%walnut shell powder); and (**f**) WSPC resin-permeated parts (40% walnut shell powder).

The interfacial binding mechanism between the walnut shell powder and amorphous Co-PES matrix, as well as the dispersion of powder particles are important factors influencing mechanical properties of WSPC parts. The laser power is the main input power that combines the Co-PES powder and the walnut shell powder, and the ingredient proportion of the powder is a factor influencing the power absorption of the WSPC powder. Therefore, by SEM, the microstructure of the cross section of the WSPC parts and after-treatment parts was observed, as shown in Figure 7. It can be seen in Figure 7a,b that the walnut shell particles are evenly distributed in the Co-PES matrix, and there is no aggregation phenomenon. The size and quantity of inner pores of the WSPC parts (22% walnut shell powder) are small, sintering neck is large and sintering zone is continuous. However, the size and quantity of inner pores of the WSPC parts (40% walnut shell powder) are large, sintering neck and sintering zone are small. Due to the small content of walnut shell powder, Co-PES powder can fully absorb the power of the laser radiation and melt completely, and its liquidity improves. Then, the bonded and wrapped walnut shell powder causes many continuous sintering zones. However, when the content of walnut shell powder is large, the Co-PES powder is prevented from absorbing the laser power, and the Co-PES powder liquidity declines. Therefore, the size and quantity of its inner pores is small and sintering zone is small.

To reduce the quantity of inner pores of the WSPC parts and improve the performance of the WSPC parts, WSPC parts are processed by permeating wax and permeating resin. The cross-section morphologies of after-treatment parts are shown in Figure 7c–f. The size and quantity of inner pores of the wax-permeated parts (22% walnut shell powder) are large, and interface bonded loosely. However, the size and quantity of inner pores of the wax-permeated parts (40% walnut shell powder) are small and the interface bonded tightly. The main reason is that, when the content of walnut shell powder is small, internal structure of WSPC parts is dense; thus, it is not easy for liquid wax to permeate to the inside of parts and fill the inner pores. However, when the content of walnut shell powder is large, internal structure of WSPC parts is loose; thus, it is easy for liquid wax to permeate to the inside of parts and fill the inner pores. Therefore, better wax-permeated parts are obtained when the large walnut shell content; the size and quantity of inner pores of the resin-permeated parts (22% walnut shell powder) are large, and the sintering neck is seen clearly. The size and quantity of inner pores of the resin-permeated parts (40% walnut shell powder) are small, and the inner structure is dense. When the content of walnut shell is small, internal structure of WSPC parts is dense, thus, it is not easy for liquid resin to permeate to the inside of WSPC parts and fill the inner pores. However, when the content of walnut shell powder is large, internal structure of WSPC parts is loose. Thus, it is easy for liquid resin to permeate to the inside of WSPC parts and fill the inner pores. Therefore, better resin-permeated parts are obtained when the large walnut shell content. To sum up, the small walnut shell content leads to fewer pores and denser structure of WSPC parts. However, the influence of after-treatment is weak, especially permeating resin treatment; the large content of walnut shell leads to more pores and loose structure of WSPC parts. However, the influence of after-treatment is good, especially permeating wax treatment. The size and quantity of inner pores of WSPC parts are small, and the interface bonded tightly.

### 3.5. Surface Quality

**Figure 8:**
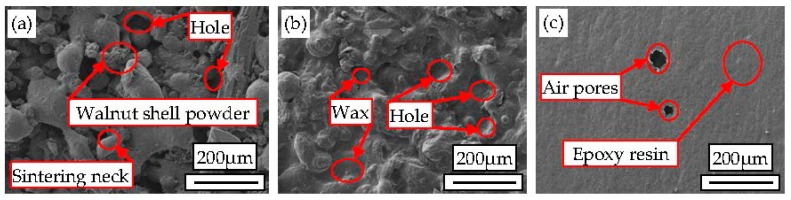
The surface morphology of WSPC parts and after-treatment parts (40% walnut shell powder): (**a**) sintered parts; (**b**) wax-permeated parts; and (**c**) resin-permeated parts.

By SLS experiment, the WSPC parts of different ingredient proportions were obtained. By after-treatment, and the wax-permeated parts and resin-permeated parts were obtained. The surface morphologies of WSPC parts, wax-permeated parts and resin-permeated parts were observed via SEM, as shown in Figure 8. Figure 8a shows the surface morphology of the WSPC parts (40% walnut shell powder). The size and quantity of inner pores of the WSPC parts are large. Co-PES powder particles are not fully melted. Sintering neck is smaller, and there is no continuous sintering zone. Due to large walnut shell content and small Co-PES powder content, molten Co-PES powder are unable to bond and wrap walnut shell powder particles. The other reason is the walnut shell powder content is large, which prevents the laser power from radiating on Co-PES powder particles. Thus, the power of laser radiation is not fully absorbed by Co-PES powder particles. Figure 8b shows the surface morphology of the WSPC wax-permeated parts (40% walnut shell powder). The size and quantity of inner pores are small but many holes on the surface, causing the surface out of flatness. Its main reason is that the surface pores are filled with liquid wax, which makes the size and quantity of inner pores are small. However, the liquidity of liquid wax is good, permeating to the inner of parts through the pores, thus there are many holes on the surface of wax-permeated parts. Figure 8c shows the surface morphology of the resin-permeated parts (40% walnut shell powder). There is almost no pore on the surface of resin-permeated parts, and surface is flat but a few holes. Its main reason is that, the surface pores are filled with liquid resin. The liquidity of liquid resin is poor, but viscosity is good, which leads to an adhesive film on the surface of parts. The air pores are produced by bonding and solidification during liquid resin immersing in the inside of parts. To sum up, the surface quality of wax-permeated parts and resin-permeated parts is superior to WSPC parts, especially, the surface quality of resin-permeated parts is best.

### 3.6. Mechanical Properties

**Figure 9:**
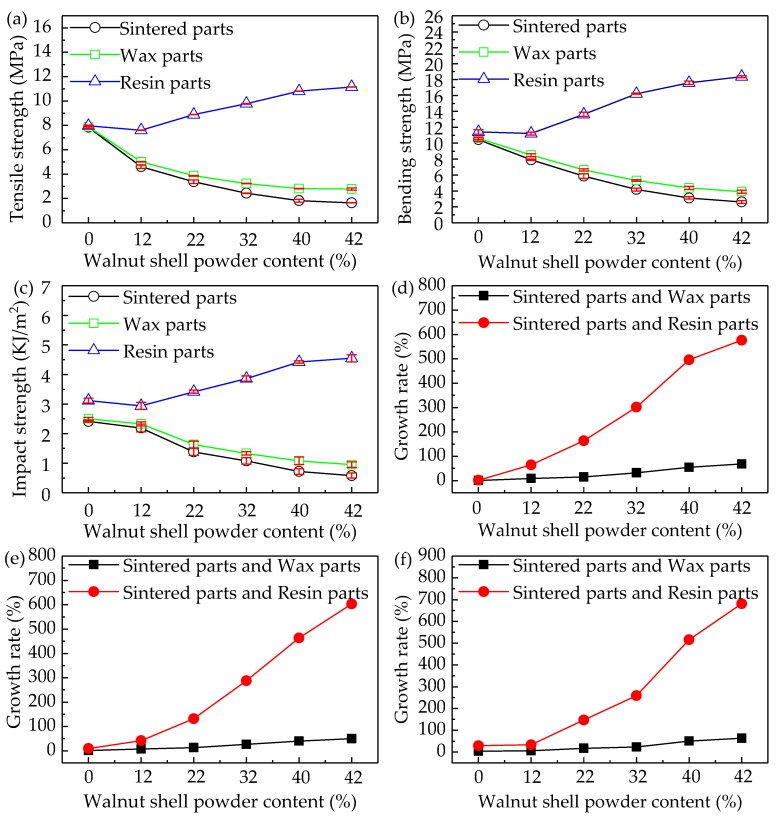
The mechanical properties change and growth rate curves of sintered parts and after-treatment parts with different ingredient proportions: (**a**) tensile strength; (**b**) bending strength; (**c**) impact strength; (**d**) tensile strength growth rate; (**e**) bending strength growth rate; and (**f**) impact strength growth rate.

In the process of SLS, the mechanical properties are one of the criteria evaluating the quality of sintering materials and determining the performance and application range of sintering parts. The mechanical properties change and growth rate curves of WSPC parts and after-treatment parts with different ingredient proportions are shown in Figure 9. In Figure 9a–c, with the increase of walnut shell powder content, the tensile, bending and impact strength of WSPC parts and wax-permeated parts decrease. When the walnut shell powder content reaches 40%, the mechanical strengths decrease slowly and tend to be constant, their values of 1.813 MPa, 3.12 MPa, 0.718 J/m^2^, and 2.809 MPa, 4.38 MPa and 1.079 KJ/m^2^, respectively. However, mechanical strengths of resin-permeated parts decrease first, then increase, when volume fraction of the walnut shell powder is 12%, its mechanical strengths reaches the lowest, with values of 7.605 MPa, 11.21 MPa and 2.941 KJ/m^2^. When walnut shell powder content is 40%, mechanical strengths increase slowly then tend to be constant, with values of 10.811 MPa, 17.6 MPa and 4.423 KJ/m^2^.This is mainly because with the increase of the content of walnut shell powder, the Co-PES powder binder content decreases. Although fully melted, walnut shell powder particles cannot be fully bonded. The poor interface bonding strength of walnut shell powder particles and Co-PES powder binder causes the mechanical strengths of WSPC parts decrease. However, when walnut shell powder content reaches 40%, the inner interface bonding strength of walnut shell powder is poorest. Although walnut shell powder content continues increasing, the effect on mechanical strengths is very weak, almost negligible. Therefore, mechanical strengths tend to be constant finally. After the pores are filled with wax, permeating wax and Co-PES binder wrap the walnut shell powder particles completely so that the interface bonding strength WSPC parts is better. When the walnut shell powder content reaches 40%, wax in the WSPC parts plays an important role, and the mechanical strength of wax-permeated parts almost no longer change and mechanical strength curve tends to constant. The process of resin-permeated parts is the same way as the wax-permeated parts. Besides, the solidification process causes internal interface bonding strength the WSPC parts rapidly increase. When the walnut shell powder content is 12%, due to the low walnut shell powder content, pores on the surface of WSPC parts are few. The liquidity of resin is poor, permeating resin content is small and the solidification of WSPC parts is weak. Compared with Co-PES parts and resin-permeated parts (more than 12% walnut shell powder), mechanical strengths of resin-permeated parts (12% walnut shell powder) is poor relatively. When walnut shell powder content reaches 40%, mechanical strengths of permeating resin are mainly determined by the mechanical property of resin. Therefore, mechanical strengths of permeating resin tend to be constant finally.

In Figure 9d–f, the growth rates of tensile, bending and impact strength of after-treatment parts are increasing continuously; however, compared with wax-permeated parts, growth rate of resin-permeated parts increases rapidly. The main reason that, although the mechanical strength of the WSPC parts after permeating wax treatment technique is improved, the permeating wax treatment process is only mechanical combination among the walnut shell powder particles, the Co-PES powder particles and wax, causing slow growth rate of mechanical strengths of wax-permeated parts. However, after the surface pores are filled with resin, permeating resin and Co-PES binder wrap the walnut shell powder particles completely. At the same time, solidification process forms chemical cross-linking reaction, causing internal interface bonding strength the WSPC parts rapidly increase. Therefore, the growth rate of mechanical strengths of resin-permeated parts increases rapidly.

## 4. Conclusions

The WSPC powder with different ingredient proportions was prepared by mechanical mixing method. The maximum of additive amount of walnut shell powder was 40% via SLS experiments. With an increase of walnut shell power content, the size and quantity of inner pores increase, the density and mechanical properties decrease continuously. Compared to Co-PES parts, the lowest density (0.897 g/cm^3^) of WSPC parts decreases by 26.78%, and the lowest mechanical strengths (1.813 MPa, 3.12 MPa, and 0.718 J/m^2^) decrease by 76.88%, 70.14%, and 70.2%, respectively. With an increase of walnut shell power content, the size and quantity of inner pores and density of after-treatment parts decrease. The mechanical properties of wax-permeated parts decrease, and those of resin-permeated parts firstly decrease, then increase. Compared to WSPC parts, the density, tensile, bending and impact strength of wax-permeated parts increase slightly by 30.32%, 54.94%, 40.36%, and 50.28%, respectively. However, those of resin-permeated parts increase considerably by 28.76%, 496%, 464%, and 516%, respectively. Therefore, a sustainable, low-cost and environmentally friendly WSPC was feasible to be used as feedstock of SLS. Moreover, the surface quality, density and mechanical properties of after-treatment parts are improved.

## Figures and Tables

**Table 1:** Volume content of ingredient in walnut shell composites (WSPC) powder.

Serial Number	Walnut Shell Powder (%)	Co-PES Powder (%)	Micro-Additive (%)
1	0	98	2
2	7	91	2
3	12	86	2
4	17	81	2
5	22	76	2
6	27	71	2
7	32	66	2
8	37	61	2
9	40	58	2
10	42	56	2
11	47	51	2
12	52	46	2
